# The intracranial pressure curve correlates to the pulsatile component of cerebral blood flow

**DOI:** 10.1007/s10877-018-0129-0

**Published:** 2018-03-16

**Authors:** Mårten Unnerbäck, Eric L. Bloomfield, Sven Söderström, Peter Reinstrup

**Affiliations:** 1 Department of Clinical Sciences Lund, Intensive Care and Perioperative Medicine, Lund University, Skane University Hospital, Malmö, Sweden; 2 Department of Anesthesiology/CCM, Mayo Clinic, Rochester, MN USA; 3 Department of Clinical Sciences Lund, Neurosurgery, Lund University, Skane University Hospital, Lund, Sweden; 4 IPV SUS Malmö, Inga Marie Nilssons gata 47, 205 02 Malmö, Sweden

**Keywords:** Cerebral blood flow, Phase contrast magnetic resonance imaging, Intracranial pressure, Mathematical analysis, Monitoring

## Abstract

Current methods to measure cerebral blood flow (CBF) in the neuro critical care setting cannot monitor the CBF continuously. In contrast, continuous measurement of intracranial pressure (ICP) is readily accomplished, and there is a component of ICP that correlates with arterial inflow of blood into the cranial cavity. This property may have utility in using continuous ICP curve analysis to continuously estimate CBF. We examined the data from 13 patients, monitored with an intraventricular ICP device determining the pulsatile amplitude ICP_amp_ as well as the area under the ICP curve (AUC_ICP_). Using an elastance measurement, the ICP curve was converted to craniospinal volume (AUC_ΔV_). The patients were examined with Phase Contrast Magnetic Resonance Imaging (MRI), measuring flow in the carotid and vertebral arteries. This made it possible to calculate CBF for one cardiac cycle (ccCBF_MRtot_) and divide it into the pulsatile (ccCBF_MRpuls_) and non-pulsatile (ccCBF_MRconst_) flow. ICP derived data and MRI measurements were compared. Linear regression was used to establish wellness of fit and ANOVA was used to calculate the *P* value. No correlation was found between ICP_amp_ and the ccICP_MRpuls_ (*P* = 0.067). In contrast there was a correlation between the AUC_ICP_ and ccCBF_MRpuls_ (R^2^ = 0.440 *P* = 0.013). The AUC_ΔV_ correlated more appropriately with the ccCBF_MRpuls_. (R^2^ = 0.688 *P* < 0.001). Our findings suggests that the pulsatile part of the intracranial pressure curve, especially when transformed into a volume curve, correlates to the pulsatile part of the CBF.

## Introduction

Patients in the neurointensive care setting often have intracranial pressure (ICP) measured in real-time, using an implantable device. Once ICP is quantified, and arterial blood pressure is measured directly from an arterial catheter, it is possible to calculate cerebral perfusion pressure (CPP) in real time as the difference between ICP and mean arterial blood pressure (MAP) (CPP = MAP − ICP). CPP is recommended to guide ICP and blood pressure management [1] because a certain CPP is thought to correlate to an appropriate CBF. Monitoring of CPP, with its concomitant therapy, seems to decrease mortality [2]. However, determining the CBF continuously with a technology that can be applied bedside, may add vital information to patient management and further improve clinical outcomes [3].

The brain is contained within a closed c ompartment consisting of the calvarium and skull base. As the content of the cranial cavity is virtually non-compressible any arterial inflow into the cranial cavity must be compensated by an outflow of venous blood and cerebro spinal fluid (CSF) [4]. While the venous blood is transported away from the cranial cavity, the CSF pressed through the foramen magnum during systole flows back at the latter part of the cardiac cycle [5–7] causing a CSF net flow over the cardiac cycle that is close to nil. The relationship between intracranial volume (ICV) and ICP was first described in the Monro-Kellie doctrine. An alteration in ICV relates to ICP in an exponential manner [8, 9]. If the relationship between intra-cranial pressure and volume is known (i.e. elastance) and the CSF contribution to this over the cardiac cycle is nil, it is possible to compute the change of intracranial- or cerebral-blood volume (CBV) during a cardiac cycle [7, 10]. The ICP curve does not directly relate to the total CBF [11], though analysis of the ICP curve morphology could identify low CBF states [12].

**Fig. 1:**
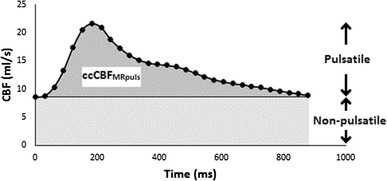
Flow measured with MRI over one cardiac cycle in one individual. Each data point represents the sum of flow in the carotid and vertebral arteries. Total AUC from one cardia cycle represents *ccCBF*_*MRtot*_ which can be divided into the non-pulsating part *ccCBF*_*const*_ and the pulsatile part *ccCBF*_*puls*_

The cerebral blood flow varies over the cardiac cycle (Fig. 1), but only the flow above the baseline, the pulsating part, could cause the rise in ICP. If the cerebral elastance is known, it should be possible to mathematically transform the ICP curve into the pulsatile part of CBF. This hypothesis can be evaluated using a CBF technique that is able to separate the pulsatile part of the CBF. Magnetic resonance imaging (MRI) using phase contrast technology has made it possible to measure flow in the four arteries supplying the brain with blood, thereby determining CBF [13]. Using this method also makes it possible to separate the pulsatile part of the CBF. In this study we compared ICP derived variables with CBF measured with cine phase contrast MRI.

## Methods and material

Ethical approval for the study was granted by the Regional Ethical Review Board at Lund University (2014/403).

All ICP data acquired at the neuro-intensive care unit (NICU) at Scania University Hospital in Lund were collected in a database. Patients admitted to the NICU during the time period August 2010—April 2013 were included in the study if they had had the ICP monitored in real time with an intra ventricular catheter, but otherwise had an intact enclosure of the cranial cavity. All patients entered into the study also were examined with cine-phase contrast MRI.

The MRI examinations were examined for quality, and suboptimal or uninterpretable examinations were excluded. The patients’ ICP curves were then examined, and only those in which a stable, non-damped curve had been recorded within 30 min after the MRI examination were included in the final data analysis.

The ICP was measured with a 8 F tunneled intraventricular catheter (HanniKath, Smiths Medical Deutschland GmbH) attached to a cerebro-spinal fluid (CSF) drainage set (HanniSet, Smiths Medical Deutschland GmbH). Removed CSF volume data were digitally registered with a Philips Intellivue MP70 STAD and stored in a database at a sampling rate of 125 Hz. The ICP pressure transducer zero was referenced to the highest point of the head, since this was clinical practice. Using this zeropoint for the ICP transducer underestimates the ICP slightly, and sometimes causes negative ICP values, but the measurements derived using this method are more consistent and reproducible [14].

The patients at the NICU at Scania University Hospital have (on a regular basis) CSF aspirated through the ventricular ICP catheter for culture and cell count. Data of the ICP before and after this aspiration and the exact volume aspirated from the aspiration closest in time to the MRI examination were used for calculation of elastance.

The ICP curve has previously been described as a function of the natural logarithm in variations of the expression:

1 $${\text{ICP}}={\text{IC}}{{\text{P}}_{{\text{eq}}}} \times ~{{\text{e}}^{{\text{E}}~ \times ~\Delta {\text{V}}}}$$

where ${\text{IC}}{{\text{P}}_{{\text{eq}}}}$ is defined as the normal, physiological steady state ICP before the change in intracranial volume (ΔV), and E is the elastance coefficient [8–10, 15, 16].

Using this expression the change in ICP (ΔICP), as a function of ΔV over the intracranial pressure cycle, can be calculated by rewriting the equation:

2 $$\Delta {\text{ICP}}={\text{IC}}{{\text{P}}_0}\left( {{{\text{e}}^{{\text{E}}~ \times ~\Delta {\text{V}}}} - 1} \right)$$

where ICP_0_ is the ICP at the beginning of the intracranial pressure cycle [10].

To determine intracranial elastance, the equation can be rewritten as:

3 $${\text{E}}=~\frac{1}{{\Delta {\text{V}}}}~ \times \ln \left( {\frac{{\Delta {\text{ICP}}}}{{{\text{IC}}{{\text{P}}_0}}}+1} \right)$$

The change in intra cranial volume (ΔV) may be calculated by another rewriting of the equation:

4 $$\Delta {\text{V}}=~\frac{1}{{\text{E}}}~ \times \ln \left( {\frac{{\Delta {\text{ICP}}}}{{{\text{IC}}{{\text{P}}_0}}}+1} \right)$$

All calculations were made using the measured ICP values, adding 15 mmHg. This has to be done since formula III is unsolvable if the ICP goes from a positive to a negative value after CSF extraction. 15 mmHg was chosen before calculations were done since it was our experience that this would adjust measured negative values to positive values in all patients without overcompensation.

In each individual, intracranial elastance was determined using equation III and the data from the CSF extraction. ICP_0_ was defined as the lowest diastolic ICP on the trend curve before extraction of CSF. ΔICP was defined as the difference between the lowest diastolic ICP on the trend curve before and after extraction.

The first stable ICP curve recorded after the MRI examination was used. The ICP pulse pressure amplitude (ICP_amp_) was calculated by subtracting the diastolic ICP from the maximum systolic ICP. The area under the ICP pulse curve (AUC_ICP_) was calculated by integrating the ICP curve over the time of one cardiac cycle. By applying formula IV and the elastance determined from equation III, the ICP curve was converted to a ΔV curve. This was then integrated over the time of one cardiac cycle, resulting in the area under this volume curve (AUC_ΔV_).

All MRI examinations were made using a Philips Intera 3.0 T magnet. Slices were 6 mm thick and a 256 × 128 matrix was used. The velocity encoding value was set to 90 cm/s, and a flip angle was set to 15°. TR was 26 ms. Each cardiac cycle was sampled at 35 time points, and the total examination time was 2 min.

The MRI examinations were analyzed using the freely available software: SEGMENT v 1.9 R3763 [17]. One examiner, masked to the ICP curves, analyzed the examinations [18]. The region of interests, ie, the internal carotid arteries and the vertebral arteries, were identified and the flow in these arteries were acquired pixel by pixel. The total flow in the carotid and vertebral arteries was summarized at each measuring point over the cardiac cycle, yielding the total flow at that point. The CBF over one cardiac cycle (ccCBF_MRtot_) is the AUC of this flow curve. The pulsatile part (ccCBF_MRpuls_) equals the ccCBF_MRtot_ minus the non-pulsatile part (ccCBF_MRconst_). (Fig. 1) ccCBF_MRpuls_ was compared to ICP_amp_, AUC_ICP_ and AUC_ΔV_ in each individual.

All statistical analysis was performed using IBM SPSS Statistics for Windows, Version 22.0. (IBM Corp, USA) Linear regression was used to establish goodness of fit, ANOVA was used to calculate the *P* value. A *P* value of < 0.05 was considered statistically significant. All values are presented as mean ± SD, unless stated otherwise.

## Results

A total of 24 patients were identified in the database. 11 patients were excluded due to suboptimal examinations. Of these 11, in 8 patients, no reliable ICP curve had been electronically registered in adjunct to the MRI, and in 3 patients the MRI examination had not been obtained at a level appropriate for measurement of CBF.

The 13 included patients had a mean age of 50 ± 15 years, range 22–75 years. Five patients were female and eight male. Patient data are summarized in Table 1.

**Table 1:** Individual patient data

Age—sex	Diagnosis	ICP_mean_ (mmHg)	CPP (mmHg)	CBF (ml/min)	ccCBF_MRpuls_ (ml)	ICP_amp_ (mmHg)	AUC_IPC_ (mmHg s)	AUC_ΔV_ (ml s)
66 years—F	ICH	1.92	81	540	4.105	15.44	3.50	1.58
56 years—F	SAH	6.40	73	664	3.872	4.88	0.83	1.23
55 years—F	SAH	6.21	94	1016	4.107	5.81	1.17	0.64
22 years—M	TBI	14.65	69	711	4.755	8.44	2.39	1.44
44 years—M	HC	− 5.22	88	537	1.693	2.50	0.65	0.59
76 years—F	CH	1.76	127	810	3.584	4.13	1.38	0.69
34 years—M	TBI	11.68	57	1007	5.523	7.06	1.77	1.44
28 years—M	TBI	11.14	66	799	7.176	8.13	2.71	1.74
38 years—M	SAH	12.69	57	976	5.817	11.75	4.66	1.76
54 years—M	TBI	3.06	85	741	3.707	4.19	0.99	1.04
64 years—M	SAH	8.97	84	811	5.042	8.56	2.51	1.54
52 years—M	CH	8.39	93	996	4.664	7.75	2.69	1.07
63 years—F	SAH	11.53	98	725	2.469	5.0	0.89	0.52

Age in years

*F* female or *M* male, *ICH* intracerebral hemorrhage, *SAH* subarachnoidal hemorrhage, *TBI* traumatic brain injury, *HC* hydrocephalus, *CH* cerebellar hemorrhage, *ICPmean* mean intracranial pressure, *CPP* cerebral perfusion pressure, *CBF* cerebral blood flow, *ccCBFpuls* the pulsatile part of the MRI measured CBF over one cardiac cycle, *ICP*_*amp*_ ICP pulse amplitude, *AUC*_*ICP*_ area under the pulsatile part of the ICP curve, *AUC*_*ΔV*_ area under the pulsatile change in volume

Intracranial elastance was 0.083 ± 0.034 ml/mmHg. The ICP_amp_ was 7.202 ± 3.350 mmHg, the AUC_ICP_ curve was 2.012 ± 1.152 mmHg s, and the AUC_ΔV_ was 1.178 ± 0.432 ml s. The ccCBF_MRpuls_ was 4.347 ± 1.364 ml.

**Fig. 2:**
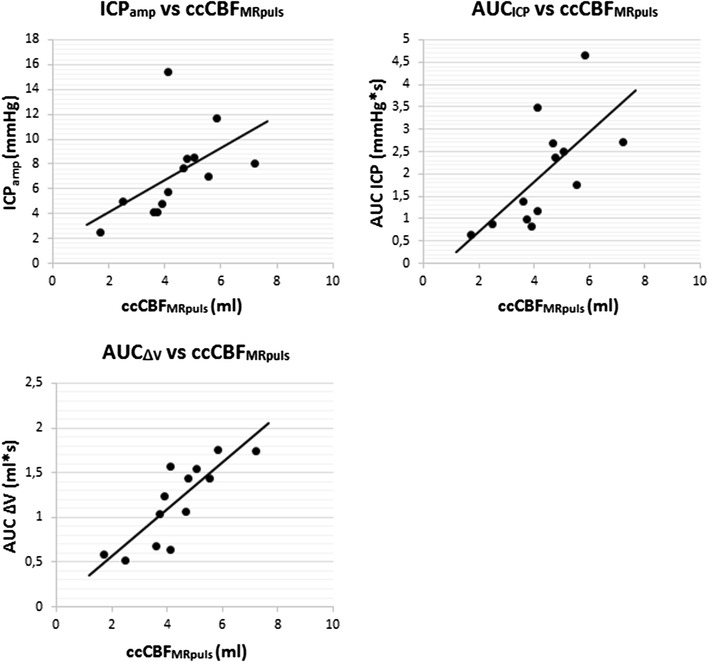
Linear regression plots of the different ICP derived values against one MRI measured cardiac cycle pulsatile AUC of CBF (ccCBF_MRpulse_): ICP amplitude (*ICP*_*amp*_) versus *ccCBF*_*MRpuls*_, area under the pulsatile ICP curve (*AUC*_*ICP*_) versus (*ccCBF*_*MRpuls*_) and area under the pulsatile change in volume (*AUC*_*ΔV*_) versus *ccCBF*_*MRpuls*_

Linear regression models of the ccCBF_MRpuls_ versus the ICP_amp_, AUC_ICP_ and AUC_ΔV_ are summarized in Table 2 and plotted in Fig. 2. There was no significant correlation between ccCBF_MRpuls_ and ICP_amp_ (*P* = 0.067). AUC_ICP_ and AUC_ΔV_ were significantly correlated to ccCBF_MRpuls_. (*P* = 0.013 and *P* < 0.001 respectively).

**Table 2:** Linear regression model of the pulsatile part of the MRI measured CBF (*ccCBF*_*MRpuls*_) vs ICP pulse amplitude (*ICP*_*amp*_), and area under the pulsatile part of the ICP curve (*AUC*_*ICP*_) and area under the pulsatile change in volume (*AUC*_*ΔV*_)

CBF_puls_ (n = 13)	R^2^	Coefficient	95% CI	Adjusted R^2^	*P* value
ICP_amp_	0.2738	1.2848	− 0.1039–2.6735	0.2077	0.067
AUC_ICP_	0.4402	0.5605	0.1411–0.9799	0.3894	0.013
AUC_ΔV_	0.6878	0.2629	0.1453–0.3804	0.6594	< 0.001

These results are plotted in Fig. 2

*R*^2^ coefficient of determination, *95% CI* 95% confidence interval

## Discussion

If the venous outflow from the cranial cavity is constant, the change in cerebral blood volume should correlate directly to the arterial cerebral inflow. However, there have been controversies regarding the cerebral venous outflow. Investigation using Doppler technique suggests that the cerebral venous outflow is almost constant over the cardiac cycle [19]. If the rise in ICP is determined by the pulsating inflow of arterial blood and the venous outflow is constant, the change in intracranial volume can be calculated using formula I. Under the condition of a constant venous cerebral outflow, the ICP_amp_ was found to correlate to the amplitude of the cerebral arterial blood volume, which correlated to the arterial inflow [20]. The rational being that a higher arterial pulse pressure would produce a higher intracranial pulse volume and a higher pulse increase in ICP. In our study such a connection was not found when comparing the ICP_amp_ and the pulsatile CBF measured with MRI or ccCBF_MRpuls_. One explanation could be that a lower flow over a longer period of time would produce a curve with lower amplitude. This happens despite the total volume being the same as in the situation of a higher flow over a shorter period of time. AUC_ICP_ should take this into account and using the AUC_ICP_ rather than the mere amplitude correlated better with ccCBF_MRpuls_. This strengthens the hypothesis that the ICP curve is partly dependent on the pulsatile arterial inflow.

The volume–pressure relationship differs between patients. Recalculating the ICP curve into a ΔV curve, using formula IV, takes the individual’s elastance and the nonlinear relationship between ICP and ICV into account. Comparing the AUC_ΔV_ with ccCBF_MRpuls_ resulted in an improved correlation compared to AUC_ICP_. This finding supports the validity of the mathematical models previously developed in animal studies [10] for the use in human subjects.

Early animal studies have estimated the change in cerebral blood volume over the cardiac cycle based on the ICP curve. Evidence was found for a pulsatile cerebral venous outflow [10] in agreement with MRI phase-contrast technology studies in humans [21]. Since cerebral blood volume is determined by both arterial inflow and venous outflow, it could be argued that the correlation between cerebral blood volume and arterial inflow is unclear. The cerebral veins are thin-walled and by consequence highly affected by the ICP. As a result the venous outflow accelerates as the ICP rises due to the pulsatile arterial influx. This relationship between ICP and venous outflow should follow the relationship between pressure and volume in equation I. Since the venous outflow must equal the arterial inflow over the cardiac cycle [4] the AUC of the venous outflow curve should correlate to the arterial inflow. This would explain the correlation between AUC_ΔV_ and ccCBF_MRpuls_ despite the pulsatile venous outflow.

To determine the CBF over the cardiac cycle, we used the MRI cine phase contrast technique. The method is well described and produces absolute values (ml/s). The technique depends on the fact that there is a phase shift proportional to the velocity when moving spins travels in a magnetic field gradient. Hence the flow velocity can be measured and by multiplying it with the arterial cross section area, the flow in each artery can be calculated. By measuring the flow in the internal carotid as well as the vertebral arteries the total CBF can be measured [13]. Validating studies of the technique has reported methodological errors of max 10% [13, 22]. To our knowledge there has been no comparison study between the arterial measurement with phase contrast MRI and other more clinically used methods; though flow velocity of the superior sagittal sinus has been shown to correlate significantly to CBF measured with ^133^Xenon clearance method [23].

Using MRI technology for measurement of CBF allows us to separate the ccCBF_MRtot_ into ccCBF_MRconst_ and ccCBF_MRpuls_. Since only the pulsatile part of the cerebral blood flow could generate a change in volume compared to baseline, the ccCBF_MRpuls_ should be the variable correlated to the ICP curve.

ICP recording was not possible in the MRI lab at our facility. Therefore the CBF values measured in the MRI lab were compared to an ICP not recorded simultaneously but within 30 min. This could produce errors due to the fact that the patient could be at a different physiological state at the two times and a simultaneous measurement would perhaps have yielded a more robust result.

Due to the reference point used in the ICP measurements the values had to be adjusted. The correction used was predefined, but the calculated AUC_ΔV_ is to some extent dependent on the chosen value. Using this reference point should however minimize errors due to uncertain reference levels for the ICP transducer, which also would affect the AUC_ΔV_ calculations.

The calibration process, determining the elastance, is of extreme importance in the computation of AUC_ΔV_. The method is based on extraction of a volume of cerebrospinal fluid, and registration of the changes in ICP. The extraction of the volume is necessarily performed over several seconds, making it possible for more slow reacting compensatory mechanism to take place compared to the physiological rapid changes over a cardiac cycle [24, 25]. This probably reduces the measured *∆*ICP compared to a more rapid change of intracranial volume. Injection of a volume with a flow corresponding to the normal physiological situation could probably yield a more robust calibration; but this may be a threat to patient safety. Although these questions remain, it is clear from our findings, that the present elastance values strengthened the correlation between ccCBF_MRpuls_ and AUC_ICP_ by recalculating the latter to AUC_ΔV_. For how long the calibration is valid remains unclear. Factors that can change the elastance are mainly changes in non-venous intra-cranial volume. These would include brain volume, CSF volume or hemorrhage, or changes in the central venous pressure.

The increase in ICP causes the CSF to flow out of the cranial cavity through the foramen magnum, but in contrast to the venous flow, it flows back into the cranial cavity. The return of the CSF occurs as the ICP falls at the end of the cardiac cycle and the net flow over the cardiac cycle is negligible [5–7, 26–28]. It does however affect the venous volume which is transported out from the cranial cavity by the increase in ICP.

Since the relationship between ICP and volume has been described as logarithmic [8–10, 15, 16], the displaced volume, venous blood and CSF is not linearly related to the ICP. Formula IV describes this relationship and applying this to the ICP curve tells us that less CSF and venous blood is displaced per pressure unit in the initial phase of the ICP curve, when ICP is relatively high, than in the latter phase, when ICP is relatively low. Although the net volume of CSF displaced over the cardiac cycle is negligible it affects the correlation between ccCBF_puls_ and AUC_ICP_. As described above, less CSF is displaced due to the relatively high ICP in the initial phase of the ICP curve than in the later phase. Calculating the AUC_ΔV_, using formula IV, taking the elastance and the logarithmic relationship into account should correct this, thereby neutralizing the effect of CSF flow on the relationship between ICP and ccCBF_puls_.

## Conclusion

In conclusion we found a significant correlation between the AUC_ICP_ and the ccCBF_MRpuls_ measured with cine phase contrast MRI. The AUC_ΔV_ calculated by using elastance and the ICP curve had a stronger correlation to the ccCBF_MRpuls_.

The correlation supports previous hypotheses that the ICP curve morphology is, at least to some extent, caused by cerebral blood flow changes. The finding raises the possibility that the mathematical analysis of the ICP curve could be useful in determining the pulsatile part of CBF bedside in real time.
